# Deposition distribution of the new coronavirus (SARS-CoV-2) in the human airways upon exposure to cough-generated droplets and aerosol particles

**DOI:** 10.1038/s41598-020-79985-6

**Published:** 2020-12-31

**Authors:** Balázs G. Madas, Péter Füri, Árpád Farkas, Attila Nagy, Aladár Czitrovszky, Imre Balásházy, Gusztáv G. Schay, Alpár Horváth

**Affiliations:** 1 Environmental Physics Department, Centre for Energy Research, Budapest, Hungary; 2 Laser Applications and Optical Measurement Techniques, Applied and Nonlinear Optics, Institute for Solid State Physics and Optics, Wigner Research Centre for Physics, Budapest, Hungary; 3 Envi-Tech Ltd., Budapest, Hungary; 4 Department of Biophysics and Radiation Biology, Semmelweis University, Budapest, Hungary; 5 Department of Pulmonology, County Institute of Pulmonology, Törökbálint, Hungary; 6 Medical Department, Chiesi Hungary Ltd., Budapest, Hungary

**Keywords:** Respiration, Viral infection, Infection, Fluid dynamics

## Abstract

The new coronavirus disease 2019 (COVID-19) has been emerged as a rapidly spreading pandemic. The disease is thought to spread mainly from person-to-person through respiratory droplets produced when an infected person coughs, sneezes, or talks. The pathogen of COVID-19 is the severe acute respiratory syndrome coronavirus 2 (SARS-CoV-2). It infects the cells binding to the angiotensin-converting enzyme 2 receptor (ACE2) which is expressed by cells throughout the airways as targets for cellular entry. Although the majority of persons infected with SARS-CoV-2 experience symptoms of mild upper respiratory tract infection, in some people infections of the acinar airways result in severe, potentially fatal pneumonia. However, the induction of COVID-19 pneumonia requires that SARS-CoV-2 reaches the acinar airways. While huge efforts have been made to understand the spread of the disease as well as the pathogenesis following cellular entry, much less attention is paid to how SARS-CoV-2 from the environment reach the receptors of the target cells. The aim of the present study is to characterize the deposition distribution of SARS-CoV-2 in the airways upon exposure to cough-generated droplets and aerosol particles. For this purpose, the Stochastic Lung Deposition Model has been applied. Particle size distribution, breathing parameters supposing normal breathing through the nose, and viral loads were taken from the literature. We found that the probability of direct infection of the acinar airways due to inhalation of particles emitted by a bystander cough is very low. As the number of viruses deposited in the extrathoracic airways is about 7 times higher than in the acinar airways, we concluded that in most cases COVID-19 pneumonia must be preceded by SARS-CoV-2 infection of the upper airways. Our results suggest that without the enhancement of viral load in the upper airways, COVID-19 would be much less dangerous. The period between the onset of initial symptoms and the potential clinical deterioration could provide an opportunity for prevention of pneumonia by blocking or significantly reducing the transport of viruses towards the acinar airways. Therefore, even non-specific treatment forms like disinfection of the throat and nasal and oral mucosa may effectively keep the viral load of the upper airways low enough to avoid or prolong the progression of the disease. In addition, using a tissue or cloth in order to absorb droplets and aerosol particles emitted by own coughs of infected patients before re-inhalation is highly recommended even if they are alone in quarantine.

## Introduction

The new coronavirus disease 2019 (COVID-19) has been emerged as a rapidly spreading pandemic^1^ originating from Wuhan, China^2^. There are currently few studies that define the pathophysiological characteristics of COVID-19, and there is great uncertainty regarding its mechanism of spread^3^. However, the disease is thought to spread (1) mainly from person-to-person, (2) mainly through respiratory droplets produced when an infected person coughs, sneezes or talks (3) which can land in the mouths or noses of people who are nearby or possibly be inhaled into the lungs^4^. Virological assessment of COVID-19 also suggests that the transmission is droplet-, rather than fomite-, based^5^.

The pathogen of COVID 19 is the severe acute respiratory syndrome coronavirus 2 (SARS-CoV-2)^6^, which infects the cells binding to the angiotensin-converting enzyme 2 receptor (ACE2)^7^ primarily in the respiratory system. Cells expressing ACE2 can be found throughout the airways^8^, and therefore, cellular entry of SARS-CoV-2 can also take place throughout the airways^9^. Although the majority of persons infected with SARS-CoV-2 experience symptoms of mild upper respiratory tract infection, in some people infections of the acinar airways result in severe pneumonia potentially leading to significant hypoxia with acute respiratory distress syndrome (ARDS) and death. However, the induction of COVID-19 pneumonia and ARDS requires that SARS-CoV-2 reaches the lower airways.

While huge efforts have been made to understand the spread of COVID-19 disease as well as its pathogenesis following cellular entry of SARS-CoV-2, much less attention is paid to how viruses from the environment reach the receptors of the target cells in the respiratory system. The aims of the present study are to quantify the deposition distribution of cough-generated droplets and aerosol particles carrying SARS-CoV-2, to estimate the amount of deposited SARS-CoV-2 in different parts of the human airways upon exposure to cough generated droplets and aerosol particles, and to discuss its consequences on the pathogenesis of COVID-19.

## Methods

For this purpose, the most recent version of the Stochastic Lung Deposition Model has been applied^10^. It was originally developed by Koblinger and Hofmann^11,12^, and continuously extended during the last three decades^13,14^. The concept and algorithms of the model are comprehensively described in the original publication^11^. However, a summary of the model is provided below.

Particle deposition in the extrathoracic airways is computed using empirical formulae based on particle deposition measurements in hollow airway casts. In this work, the formula of Yu et al.^15^ was used to account for the inertial deposition, while deposition due to diffusion was computed by Cheng’s equation ^16^.

Deposition in the intrathoracic airways is simulated by reconstructing the path of inhaled particles in a stochastic lung structure. Stochastic means that airway lengths, diameters, branching angles and gravity angles of the airways along the path of an inhaled particle is selected randomly from distributions of these parameters obtained by statistical analysis of morphometric data measured by Raabe et al.^17^. The geometry of the acinar airways was taken from the description of Haefeli-Bleuer and Weibel^18^.

Particles are tracked from inhalation until they deposit or leave the airways by exhalation. During inhalation, the path of the particles at bifurcations is decided by so-called entrance probabilities. At the entrance of the lobes (lobar bronchi), the entrance probability is proportional to the lobar volume. In all other bifurcations, the entrance probability is proportional to the cross sectional area of the daughter branch. In the acinar part of the airways, the particles enter the alveoli based on entrance probability which is proportional to air volume entering the alveolus divided by air volume entering the corresponding acinar duct.

Throughout their path, particles can deposit by impaction, sedimentation, and diffusion mechanisms. In the Stochastic Lung Deposition Model, the probability of deposition by each mechanism is calculated by the formulae Yeh and Schum^19^ provided for cylindrical tubes (bronchus) and spherical space (alveolus).

The main input data of the model are spirometric (functional residual capacity) and inhalation parameters (inhaled volume, inhalation time, breath-hold time between inhalation and exhalation, exhalation time, breath-hold after exhalation) and particle properties (particle density, particle size or size distribution).

The model computes the fraction of inhaled particles that deposit in each anatomical region of the lungs. In addition, it also provides the deposition fraction as the function of airway generation number. (The first airway generation consists of the trachea and the first half of the main bronchi. The second airway generation consists of the second half of the main bronchi and the first half of their daughters, and so on.) The fraction of inhaled mass in different anatomical regions and airway generations can also be obtained, which is particularly useful if the average virus concentration in the material coughed (including aerosol particles and droplets) is supposed to be independent on particle size. These fractions are quantified for a full breathing cycle (including inhalation, exhalation, and two breath holds) supposing normal breath through the nose.

Functional residual capacity of 3300 cm^3^, tidal volume of 750 cm^3^, and breathing frequency of 12 min^−1^ were taken from the Human Respiratory Tract Model of the International Commission on Radiological Protection corresponding to an adult man in sitting position^20^ and applicable for normal breath if the spine is in vertical position. It was supposed that the duration of inhalation is 1.9 s followed by 0.1 s breath hold, and exhalation lasts for 2 s followed by a 1-s-long breath hold.

**Figure 1:**
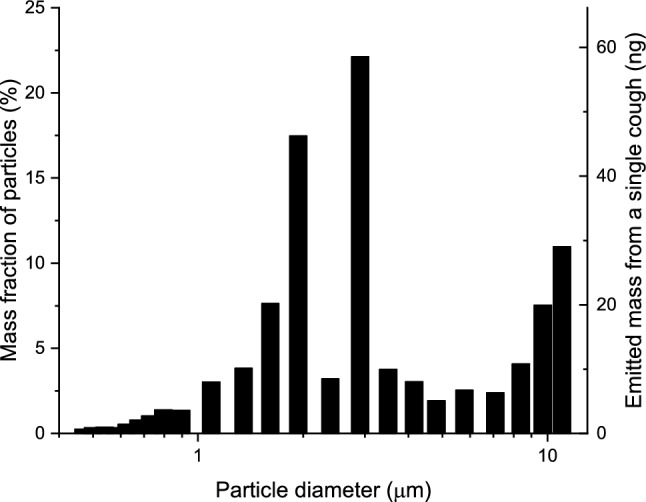
Mass size distribution of particles emitted by coughing of patients with influenza^21^. This mass size distribution was used as input of the Stochastic Lung Deposition Model.

Besides the geometry and flow conditions in the airways, lung deposition of droplets and aerosol particles is determined by their aerodynamic properties which depends on many parameters (e.g. size, shape, morphology, density etc.). Lindsley et al. measured the number size distributions of cough-generated droplets and aerosol particles by means of a combined wide range aerosol particle spectrometer (optical particle counter and scanning mobility particle sizer system), when patients with influenza cough^21^. While influenza is a different disease than COVID-19, studies of cough aerosols from patients with various respiratory infections have shown striking similarities in aerosol size distributions^22^, therefore we used the data from Lindsley et al.^21^. The mass size distribution was obtained from the concentration, number size distribution, and density of cough-generated particles. For the calculations, we assumed that the particles are spherical and their physical size is equal to their measured optical size. The mass size distribution is plotted in Fig. 1.

The cough-generated particles may travel great distance if the meteorological conditions (air velocity, temperature, humidity) are favorable^23^. During the flight, the particles may evaporate or fall down by gravitational settling. Therefore, the present simulations apply only to one of the major transmission routes, when a bystander subject (within a 1-m-distance) directly inhales cough-generated droplets and aerosol particles^4^. If the inhalation takes place more distant from the source in space or time, then the size distribution has to be measured near the subject inhaling the emitted particles.

In a recent study, it was found that viral load quantified as the number of RNA copies per cm^3^ varies highly in throat swab and sputum samples of SARS-CoV-2-infected patients with a maximum viral load exceeding 10^11^ RNA copy per cm^3^, while the median values are in the order of 10^5^–10^6^ RNA copy per cm^3^^[24]^. Values along this wide range were used to obtain upper estimate for RNA copy number deposited in different lung regions and airway generations. Other studies provided similar range for viral loads^5,25^. It is important to note, however, that the number of viruses capable of infection is much lower than the number of RNA copies measured. In another study, infectious viruses could not be isolated from samples that contained less than 10^6^ RNA copy per cm^3^^[5]^.

We supposed that the average virus concentration (concentration of viruses capable of infection) is independent on particle size. It means that the average virus number in a particle (droplet or aerosol particle) is directly proportional to the mass of the particle. It is an appropriate approximation only in locations close to the source, because evaporation increases the virus concentrations in droplets and aerosol particles, and evaporation rate strongly depends on the particle size.

### Ethics committee approval

Neither human, nor animal subjects were involved in this study, and therefore ethics committee approval was not required for this study.

## Results

**Figure 2:**
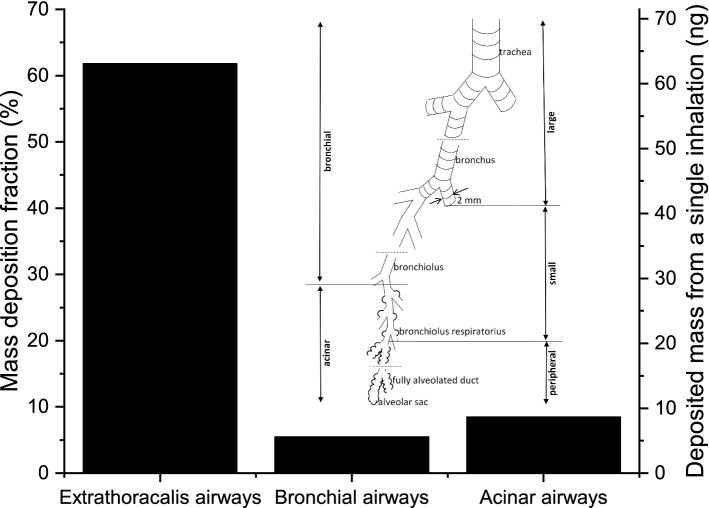
Mass deposition fraction of inhaled particles (left axis) and deposited mass from a single inhalation (right axis) in the extrathoracic, bronchial and acinar regions of the lungs upon exposure to cough-generated droplets and aerosol particles. The insert shows the subdivisions of the intrathoracic airways.

As the infection with SARS-CoV-2 causes very different symptoms depending on the infected region, first we focus on the regional deposition distribution of particles emitted by coughing. Figure 2 shows that while 61.8% of the inhaled mass is filtered out by the upper airways, significant fractions deposit in the bronchial (~ 5.5%) and acinar airways (~ 8.5%). Considering the average particle number concentration in the material coughed (29,600 dm^−3^, the term *material coughed* includes aerosol particles and droplets throughout this article), the tidal volume (0.75 dm^3^), and the mean particle mass (4.6 pg), it can be calculated that a single inhalation results in 63 ng cough-generated material depositing in the extrathoracic airways, while 5.6 ng and 8.6 ng deposit in the bronchial and acinar airways, respectively.

**Figure 3:**
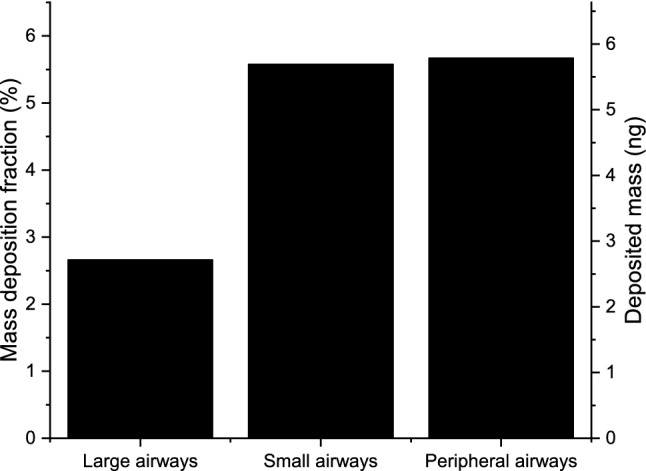
Mass deposition fraction of inhaled particles (left axis) and deposited mass from a single inhalation (right axis) in the large, small, and peripheral airways upon exposure to cough-generated droplets and aerosol particles. See also the insert in Fig. 2 for the definitions of large, small, and peripheral airways.

In a subtler subdivision of the intrathoracic airways (see also the insert in Fig. 2), one can distinguish peripheral airways (all acinar airways except the first four generations, i.e. the bronchiolus respiratorius region), small airways (with a diameter smaller than 2 mm except the peripheral airways), and large airways (with a diameter larger than 2 mm). Figure 3 shows the mass deposition fractions and deposited mass from a single inhalation in these airways. While 2.7 ng and 5.7 ng material coughed deposit in the large and small airways, respectively, 5.8 ng reach the peripheral airways, and deposit there. It may also be of interest that 2.9 ng material coughed deposits in the bronchiolus respiratorius region.

**Figure 4:**
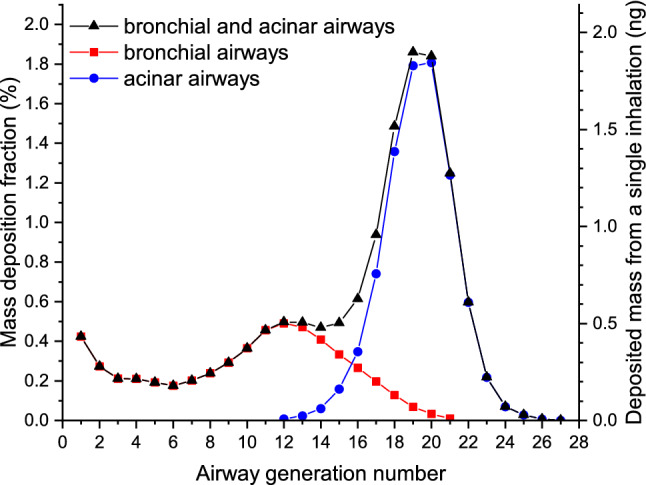
Mass deposition fraction of inhaled particles (left axis) and deposited mass from a single inhalation (right axis) upon exposure to cough-generated droplets and aerosol particles as the function of airway generation number.

Using the Stochastic Lung Deposition Model, the deposition fraction and the deposited mass as the function of airway generation number can also be determined. Figure 4 shows that in terms of deposited mass the most affected part of the acinar airways is the 19th and 20th airway generations, which means that most of the viruses penetrating the extrathoracic airways passes 19 bifurcations before depositing. In the bronchial region, the highest amount deposits in the 12th airway generation. The acinar peak is more than three-fold higher than the bronchial one. However, the difference in deposition density (deposited mass per unit surface) is much smaller as the surface of the airways strongly increases with the generation number.

In order to estimate the amount of deposited RNA copies in different parts of the airways, viral loads measured by Pan et al.^24^ was used. They found that viral loads ranged from 641 RNA copy per cm^3^ to 1.34 × 10^11^ RNA copy per cm^3^ with a median of 7.99 × 10^4^ and 7.52 × 10^5^ RNA copy per cm^3^ in throat swab and sputum samples, respectively. In case of dry coughing, viral loads in the throat have to be taken into account. Therefore, the former of the median values is considered. Using the density from Lindsey et al.^21^, these data can be converted to viral loads in unit mass resulting in values ranging from 376 RNA copy per g to 7.86 × 10^10^ RNA copy per g with a median of 4.69 × 10^4^ RNA copy per g in throat swab samples.

Combining the median value in throat swab samples with the regional deposition distribution data, it can be found that about 2500 breathing cycles are required to result in one deposited copy of RNA in the acinar airways accompanied by 7.3 RNA copies depositing in the extrathoracic, and 0.7 RNA copy depositing in the bronchial airways. Taking into account the maximal measured viral load of 1.34 × 10^11^ RNA copy per cm^3^, it can be obtained that from a single inhalation 4900 copies of RNA deposit in the extrathoracic airways, and 680 copies of RNA deposit in the acinar airways. Considering that the minimum viral load of a sample to be infectious is about 1 million RNA copy per cm^3^^[5]^, it can be concluded that the probability of direct acinar airway infection with SARS-CoV-2 from inhalation of cough-generated droplets and aerosol particles is very low.

## Discussion

Figure 1 shows that the mass size distribution of particles emitted by coughing is dominated by particles with diameters between 2 and 3 μm. Therefore, deposition of particles in this range is the major determinant of deposition distributions plotted in Figs. 2, 3, and 4. Indeed, more than half of the particles in this size range are filtered out by the extrathoracic airways by inertial impaction^26^. Particles penetrating these airways can travel far into the acinar airways resulting in a significantly higher deposition fraction in the acinar airways than in the bronchi.

Particles with a diameter smaller than 1 μm can also penetrate into the deep airways with high probability. However, most of them are ultimately exhaled. Therefore, their contribution to the total deposition fraction is significantly lower than that of the particles with a diameter between 2 and 3 μm. In contrast, particles larger than 3 μm are mainly depositing in the upper airways, and the probability that they enter the lungs decreases by their diameter. These findings are in agreement with deposition fractions obtained by five different airway deposition models (including the model applied in this study) retrieved in the open literature and compared in a review^26^.

Inhalation of particles emitted by a single cough of an individual with viral loads of 4.69 × 10^4^ RNA copy per g in the throat swab results in negligible amount of RNA copies, around 4 × 10^–4^ in the acinar airways of a bystander person. The reason for this surprising fact is that the mass of inhaled material (cough-generated droplets and aerosol particles) per inhalation is low (1.02 × 10^–7^ g), and therefore the absolute virus number is also low. We assumed homogenous virus distribution in the initial throat swab sample, and that the number of viruses in a particle is proportional to its mass. In this case, the probability that a particle contains at least one RNA copy of the virus can be calculated assuming Poisson distribution.

**Figure 5:**
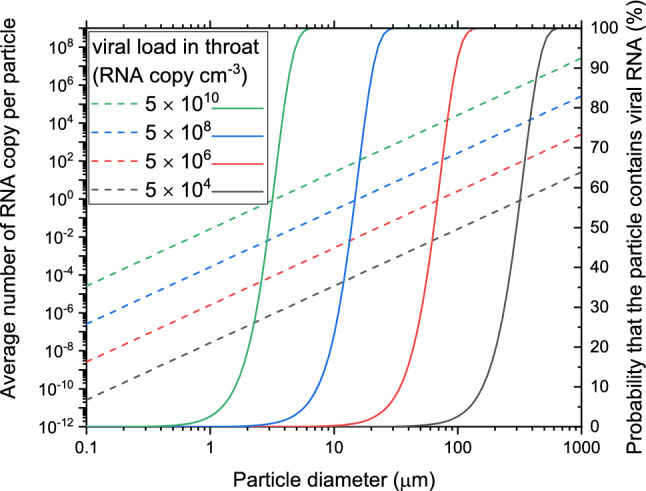
The average number of RNA copies of the virus in one particle (left axis and dash lines) and the probability that a particle contains at least one RNA copy (right axis and solid lines) as the function of particle size in case of different initial viral loads (in RNA copy per cm^3^) in the throat (different colors).

Figure 5 shows the average number of RNA copies per particle as well as the probability that a particle contains at least one RNA copy of the virus as the function of particle size. It can be seen that the average number of RNA copies in a single particle remains below 10^–4^ for particles smaller than 10 µm if the viral load in the throat swab is 5 × 10^4^ RNA copy per cm^3^. However, if the viral load is high, for example 5 × 10^10^ RNA copy per cm^3^, then the probability that a particle contains an RNA copy strongly increases from 1.3% at 0.8 µm and it is almost 100% at 8 µm, where the average number of RNA copies in the particles is more than one.

Independently on the virus concentration in the inhaled air, it can be concluded that about 7 times more viruses deposit in the extrathoracic airways than in the acinar airways. It is in agreement with the clinical observation that the typical first respiratory symptom of COVID-19 is dry cough^27,28^, which is often caused by upper respiratory infections. In addition, anosmia and ageusia have also been reported indicating infection of cells in the upper airways^5,29,30^.

Our results suggest that inhalation of cough-generated particles containing SARS-CoV-2 results directly in only an upper airway infection, which then can later develop into pneumonia. It is in agreement with the clinical observation that some patients who have only mild symptoms initially will subsequently have precipitous clinical deterioration that occurs approximately one week after symptom onset^27,28,31^. It is also in agreement with a recent virological analysis of nine mild cases of COVID-19 providing proof of active replication of SARS-CoV-2 in tissues of the upper respiratory tract^5^. They found very high concentrations of viral RNA in and isolated the virus itself from early throat swabs, although the patients had mild symptoms only. Without the enhancement of virus concentrations in the upper airways, SARS-CoV-2 would be much less dangerous. Therefore, even non-specific treatment forms like disinfection of the throat and nasal and oral mucosa (e.g. by nasal irrigation^32^ or oral rinses^33^) may effectively keep the viral load low enough to avoid or prolong the progression of the disease.

From the throat where high viral loads can be found even in patients with mild symptoms^5^, viruses can be transported either via the cardiovascular or the respiratory system. As SARS-CoV-2 genome cannot be detected in the blood of patients with mild symptoms^34^, transport via the respiratory system must be the dominant route. Potential mechanisms of virus transport from the throat to the lower airways may include re-inhalation of own cough, aerosol and droplet generation in the throat during inhalation, virus transport on the surface of the bronchial airways, or gradual infection of neighboring cells expressing ACE2 towards the periphery. As mucociliary clearance can inhibit the latter two processes, compromised mucus production or transport can be a risk factor for COVID-19 pneumonia.

Inhalation of air after a bystander cough could theoretically induce pneumonia without first developing an active infection in the extrathoracic airways, because material emitted by coughing directly reaches the acinar airways according to our models. Considering the viral load of the deposited mass, however, such direct induction of COVID-19 pneumonia requires either very large viral loads in the particles inhaled or very long exposures to particles with moderate viral loads. The latter underlines the importance of ventilation in rooms of infected people.

Another example for prolonged exposure is the continuous re-inhalation of own coughs of infected patients, which may contribute to the progression of the disease. Therefore, reducing the re-inhalation of own coughs could significantly prolong, or even block the onset of further, more severe phases of COVID-19. Therefore, using a tissue or cloth in order to absorb droplets and aerosol particles emitted by own coughs of infected patients before re-inhalation is highly recommended even if they are alone in quarantine.

### Limitations of the study

As the main tool applied in this work was a computational model, it is important to discuss the limitations of the study and the Stochastic Lung Deposition Model. The model itself has been validated earlier by comparing the deposition fractions obtained by five different airway deposition models including the Stochastic Lung Deposition Model^26^. However, the uncertainty of the results also depends on uncertainties of the input parameters we applied. In the following, effects of the input parameters (functional residual capacity and tidal volume, size distribution, and viral load of the material coughed) will be discussed.

Although, the applied functional residual capacity (3300 cm^3^) and tidal volume (750 cm^3^) were taken from the Human Respiratory Tract Model of the International Commission on Radiological Protection, it can be argued that these values are much larger than the clinicians’ daily experience. For this reason, the simulations were repeated for smaller values, for a functional residual capacity of 2300 cm^3^, and a tidal volume of 500 cm^3^. This change decreases the mass deposition fractions in extrathoracic, acinar, and peripheral airways by 10%, 18% and 26%, while increases the mass deposition fractions in the bronchial (7%), large (10%), and small airways (1%, for definitions, see the insert in Fig. 2). The decrease in the acinar and peripheral airways is stronger than the decrease in the extrathoracic airways, which confirms the conclusions of the study.

While we focused on one of the most important transmission routes of COVID-19, i.e. coughing of a bystander person, it is important to note that it is not the only way of spread of the disease, as sneeze and talk can also infect others^4^. Since the size distribution of the emitted material is different in case of cough and talk^35^, the present study is applicable for coughing only. For the validity of the results, however, it is also needed that the subject person is taking breath close (within a 1-m-distance) to the person infected directly after his or her coughing. More distant in space or time, the particle size distribution will be different due to evaporation and gravitational settling. In addition, our assumption that the virus concentration is independent on the particle size is not reasonable farther from the cough. This is because evaporation affects the virus concentration differently in particles of different sizes, which may explain why higher virus concentrations can be observed in smaller particles in general^22,36^.

The virus concentration in droplets and aerosol particles also depends on the location where the material coughed are generated. Johnson et al. identified three different processes of aerosol/droplet generation associated with three size distribution modes: bronchiolar fluid film burst, laryngeal, and oral cavity modes, with the latter two playing important roles in coughing^35^. While these modes and viral loads in the corresponding locations could have been considered in order to obtain an estimate on the average viral load in the material coughed in a more sophisticated way, it would not necessarily lead to more accurate data. Instead, we used not only the median, but also the maximal viral load measured in throat swab samples in order to cover a wide range of potential viral loads in droplets and aerosol particles. Therefore, the conclusions of the study are not weakened by the fact that the processes identified by Johnson et al.^35^ were not taken into account.

## Conclusions

In order to quantify the deposition distribution of cough-generated droplets and aerosol particles containing SARS-CoV-2, we applied the Stochastic Lung Deposition Model. It was found here that the probability of direct infection of the acinar airways due to inhalation of particles emitted by a bystander cough is very low. As the number of viruses deposited in the extrathoracic airways is about 7 times higher than in the acinar airways, we concluded that COVID-19 pneumonia must be preceded by SARS-CoV-2 infection of the upper airways in most cases. The one week difference observed in several patients between the onset of their initial mild symptoms and precipitous clinical deterioration^27,28,31^ provides a precious window for prevention of pneumonia and ARDS by blocking or significantly reducing the transport of the virus towards the acinar airways. Therefore, disinfection of the mucosa of the upper airways may help to avoid or prolong the progression of the disease. In addition, using a tissue or cloth in order to absorb droplets and aerosol particles emitted by own coughs of infected patients before re-inhalation is highly recommended even if they are alone in quarantine.

## Data Availability

The datasets generated and analyzed during the current study are available from the corresponding author on reasonable request.
